# Supplementary material for: A Comprehensive Computational Investigation into the Conserved Virulent Proteins of Shigella species Unveils Potential Small-Interfering RNA Candidates as a New Therapeutic Strategy against Shigellosis
Source: Molecules. 2022 Mar 17;27(6):1936. doi: 10.3390/molecules27061936 (PMC8950558; doi:10.3390/molecules27061936)
Supplement: Supplementary file 1 [file molecules-27-01936-s001.zip › File S1. List of conserved sequences.pdf]

File S1. List of conserved sequences.

Gene	Sequence (5'-3')
IcsA	ATGGAATGGATGCGTGGTATATAACTTCTTCCAACCCCTCTCAT
IcsA	GCATCTAGAACTAAGCTACGGATTAACTCTGATATTATGAT
IcsA	GCTGGTGATAATAATGATGGTAATAGTTGTGG
IcsA	CTATAATCAATCAAGGCATGATTCTTGGTGGTA
IcsA	GCGATAGTTATAGTGATAGTGATGGGGGGAATG
IcsA	TACCCATAATCAACAAAGGGACTATTTCC
IcsA	CCTCTCTGTAATCAATAAGGGCACGTTCGCTGGAGGCAACGGAGGTGCTGCTTAC
IcsA	TATGGTTATGATGGCTACGGTGGTAATGCTATCACAGGAGATAACCTGTCT
IcsA	AACAATGGAGCTATTTTAGGCGGTAATGGTGGACATTGGGGGGATGCTATAAATGGTAGC
IcsA	AATATGACCATTGCTAATAGCGGATATATAATTTCAGGTAAAGAAGATGATGGAACACAA
IcsA	AGGTAATGCTATCCACATCACTGGTGGAAACAATTCATTAATACTCCATGAA
IcsA	GGTTCTGTCATTACTGGTGATGTACAGGTTAACAATTCATCCATTCTGAAAATTATCAAC
IcsA	CCAACTATTGAAGGTGATTTATGTGCTGGTGATTGTACA
IcsA	AATGATTACACTGGGACCA
IcsA	ACTGTTTCACTATCAGGTAACAAATTCACTGTTTCAGGTGACGTTTCTTTTGGTGAGAAC
IcsA	AGTTCTTTAAATTTAGCTGGAATCAGTAGTC
IpaA	ATGCATAATGTAAATAATACTCAAGCG

IpaA TTCTTATATAAGGCAACTTCACCATCATCA

IpaA CAGAATACAGCGAGTTAAAAAGCAAAATATCCGATATCCATAGTTCGCAAACTTCTCTAAAAACACCA

IpaA CTTTGCAACGTCTTTTAATCAGAAATGTCTTGA

IpaA TTTTTATTTTCTTCCTCAGGGAAAGAAGATGTGTTAAGAAGCATTTATTCCAACTCAATGAATGCGTATGCCAAAAGCGAGATT

IpaA GAATTTTCAAATGTTTTGTACTCCTTAGTACATCAAAATG

IpaA
GAAAACGAAAAGGGACTTCAAAAAATTGTCGCACAGTATTCGGAACTAATTATAAAAGATAAATTATCCCAAGATTCTGCCTTTGGACCATGGTCG
GCAAAGAATAAGAAACTCCATCAATTACGACAAAACATTGAGCACAGACTTGCACTATAGCACAACAACACACATCTGGTGAAGCTTTATCATTGGGACAA
AAACTCCTCAATACTGAAGTATCATCATTTATCAAGAATAATATTCT

IpaA TAAGTAATGAAACTGTTTCATC

IpaA TTGACGCACAGGCAAAACTTGCCTTTGATAGTTTGCGCAATCAACGTAAAAATACTATTGATAGTAA

IpaA AAACTGTCAAGAGACTTAAATAC

IpaA CCTGAGCTGTTGAGAAAAGTCCTTAAT

IpaA ATATTTTAGAAGATATAAAAGATTCGCATCCTATCCAAGATGG

IpaA AAGATATGCCAGATGGCGGACCAACCCCCGG

IpaA
GCCAATGAGAAAACATCCCAACCTGTAATTCACTATCATATAAATAATGATAATAGAACTTACGATAATAGAGTTTTTGACAACAGAGTATATGAC
AATAGCTATCACGAGAACCCAGAAAATGATGCACAGTCTCCTACTTCTCAGACAAACGATCTATTA

IpaA CAAAAAGTAACTTCCGTTCTACCACACTCTATATCAGAT

IpaA CTGTCCAGACATTTGCAAATAATTCAGCTTTAGAAAAGG

IpaA TTTCAACCATACTCCAGATAATTCGGATGGAATAG

IpaA TTCAGACCTGTTAACTACGAGTAGTCAAGAAAGATCT

IpaA GGGACACAGGCCTCTGAACATACAGAACTCTT

IpaB CTTCCACTGAGCTTGGAGACAATACTATCCA

IpaB AAATGATGCAGCTAACAAATTATTTTCTCTTACAATTGCTGATCTTACTGCTAAC

IpaB
CACACTCAACTTCAAATATATTAATCCCTGAACTTAAAGCACCAAAGTCATTAAATGCAAGTTCCCAACTAACGCTTTTAATTGGAAACCTTATTCA
AATACTC

IpaB GTGAAAAATCTTTAACTGCATTAACAAATAAAATTACTGC

IpaB AAAAACCTAGAATTCTCCGATAAAATTAA

IpaB ACTCTTCTATCTGAAACTGAAGGACT

IpaB CAAATTAATAAACTAAAAAACGCAGATTCTAAAATAAAAGACCTAGAAAATAAAATTAACCAAATTCAAACAAGATTATC

IpaB
CGGGAAGAAATACAACTCACTATCAAAAAAGACGCAGCAGTTAAAGACAGGACATTGATTGAGCAGAAAACCCTGTCAATTCATAGCAAACTTAC
AGATAAATCAATGCAACTCGAAAAAGAAATAGACTCTTTTTCTGCATTTTCAAACACAGCATCTGCTGAACAGCTATCAACCCAGCAGAAATCATTAACCGG
ACTTGCCAGTGTTACTCAATTGATGGCAACCTTTATTCAACTAGTTGGAAAAAATAATGAAGAATCTTTAAAAAATGATCTGGCTCTATTCCAGTCTCTCCAA
GAATCAAGAAAAACTGAAATGGAGAGAAAATCTGATGAGTATGCTGCTGAAGTACGTAAAGCAGAAGAACTCAACAGAGTAATGGGTTGTGTTGGGAAAA
TACTTGGGGCACTTTTAACTATCGTTAGTGTTGTTGCAGCAGCTTTTTCTGGAGGA

IpaB
GTACAAGCAGCGACCGGCAATTCCTTCATGGAACAAGCCCTGAATCCGATCATGAAAGCAGTCATTGAACCCTTAATCAAACTCCTTTCAGATGCAT
TTACAAAAATGCTCGAAGGC

IpaB TGATTGGCTCTATTCTGGGGGCAATCGCAG

IpaB GTTCTCGTAGCCACTGTTGGTAAACAGGCAGCAGCAAAACTTGCAGA

IpaB TAATAGGTAAAACCCTCACAGACCTTATACC

IpaB AAGTTTCTCAAGAATTTTTCTTCTCAACTGGACGATTTAATCACTAATGCTGTTGCCAGATTAAATAAATTTCTTGGTGCAGCGGGTGATGAAGTAATATCCAAACAAATTATTTCCACCCATTTAAACCAAGCAGTTTTATTAGG

IpaB GAAAGTGTTAACTCTGCCACACAAGCGGGAGGAAGTGTCGCTTCTGCTG

IpaB ACATTATCGAAATATCAAGTTGAACAACTGTCAAAA

IpaB ATATCAGTGAAGCAATAGAAAAATTCGGCCAATTGCAGGAAGTAATTGCAGATCTATTAGCCTCAATGTCCAACTCTCAGGCTAATAGAACTGATGTTGCAAAAGCAATTTTGCAACAAACTACTGCT

IpaC TTTATATACAGATATATCCACAAAACAAACTCAAAGTTCTTCCGAAACACAAAAATCACAAAATTATCAGCAGATTGCAGCGCATATTCCACTTAATGTCGGTAAAAATCCCGTATTAACAACCACATTAAATGATGATCAACTTTTAAAGTTATCAG

IpaC GCAGGTTCAGCATGATTCAGAAATCATTGCTCGCCTTACTGACAAAAAGATGAAAGA

IpaC TTCAGAGATGAGTCACACCCTTACTCCAGAGAACACTCTGGATATTTCCAGT

IpaC TTTCTTCTAATGCTGTTTCTTTAATTATTAGTGTAGCCGTTCTACTTTCTGCTCTCCGCACTGCAGAAA

IpaC TAAATTGGGCTCTCAATTGTCATTGATTGCGTTCGATGCTACAAAATCAGCTGCAGAGAACATTGTTCGGCAAGGCCTGGCAGCCCTATCATCAAGCATTACTGGAGCAGTCACACAAGTAGGTATAACGGGTATCGGTGC

IpaC AAAAAAACGCATTCAGGGATTAGCGACCAAAAAGGAGCCTTAAGAAAGAACCTTGCCACTGCTCAATCTCTTGAAGCAGGTTCTAAATTAGGGTTAAATAAACAAATAGATACAAATATCACCTCACAAAGAGCTT

IpaC TTTTAGGTAAAAATAAACTGGCGCCAGATAATATATCCCTGTCAACTGAACATAAAACTTCTCTTAGTTCTCCCGATATTTCTTTGCAGGATAAAATTGACACCCAGAGAAGAACTTACGAGCTCAATACCCTTTCTGCGCAGCAAAAACAAAA

IpaC
ACATCAGCCGTTGCTGGTAATATATCCACATCAGGAGGGCGTTATGCATCTGCTCTTGAAGAAGAAGAACAACTAATCAGTCAGGCCAGCAGTAAACAAGCAGAGGAAGCATCCCAAGTATCTAAAGAAGCATCCCAAGCGACAAATCAATTAATACAAAAATTA

IpaC CAGCCAGTCAGATTGCTGGTAACATTCGAGCT

IpaJ ATGTCGGAACAACGGAAGCCTTGCAAACGGGGATGTATTCATACAGGTGTTATGTTATAT

IpaJ GGGGTTTTATTGCAGGGGGCTATACCAAGAGAATATATGATATCTCATCAGACTGATGTG

IpaJ AGGGTAAATGAGAATAGAGTAAACGAACAAGGTTGTTTTTTGGCGAGGAAGCAGATGTAT

IpaJ GACAATTCTTGTGGAGCTGCCAGTTTACTCTGTGCCGCAAAGGAATTAGGGGTTGATAAA

IpaJ ATACCACAATACAAAGGGTCTATGTCCGAAATGACAAGGAAGAGTTCTCTTGATTTGGAT

IpaJ AATCGTTGTGAGCGGGATTTATACTTAATAACTAGTGGTAATTATAATCCGAGAATACAC

IpaJ AAAGATAATATCGCGGATGCCGGTTATTCTATGCCTGATAAGATAGTCATGGCTACGAGG

IpaJ CTTCTTGGATTGAATGCTTATGTCGTTGAAGAAAGTAACATTTTTTCGCAAGTAATTAGT

IpaJ TTTATTTATCCCGATGCAAGGGACTTATTAATTGGCATGGGATGTAATATAGTACATCAA

IpaJ CGAGATGTGTTATCTTCTAACCAAAGAGTTTTGGAAGCTGTTGCTGTATCCTTTATTGGA

IpaJ GTTCCTGTCGGCCTCCACTGGGTGTTGTGTAGACCTGATGGAAGCTATATGGATCCAGCT

IpaJ GTAGGAGAAAATTATTCTTGTTTTTCAACTATGGAATTAGGTGCAAGAAGAAGTAATTCT

IpaJ AACTTTATAGGATATACTAAAATAGGTATTTCCATAGTTATAACTAATGAGGCTTTG

IpgB ATGCAAATTCTAAACAAAATACTTCCACAGGTTGAATTCGCGATTCCTCGCCCATCATTT

IpgB CAATAAGCTTGTAAAGAAAATATTATCTGTTTTTAACTTAAAACAA

IpgB CGCTTTCCTCAGAAGAATTTTGGATGCCCGGTGAATATAAATAAAATCAGGACA

IpgB ATAGACAAAATAAAAGATAGTAATTCGGGGAATCAACTATTTTGTTGGATGAGCCAAGAG

IpgB CTTATGTCTCTTCAATGATAAACAGAAGTATAGATGAAATGGCCATACACAAT

IpgB GGAGTTGTTTTGACCTCAGATAACAAAAAAAACATATTTGCAGCTATTGAAAAAAAATTC

IpgB CCGGATATAAAACTTGATGAAAAATCAGCACAAACATCTATCAGTCATACAGCACTAAAC

IpgB GAGATTGCCTCATCTGGCCTCAGAGCAAAGATTTTAAAACGCTATAGCAGT

IpgB TTGTTTAACACACAAATGAAGGATCTTACAAATCTAGTATCCAGTTCCGTCTACGATAAA

IpgB ATATTTAATGAATCAACAAAAGTTTTACAAATAGAAATAAGCGCAGAGGTTCTAAAGGCT

IpgB GTATACCGTCAAAGCAATACAAATTAA

IpgD ATGCACATAACTAATTTGGGATTGCATCAGGTTTCATTTCAAAGCGGAGATTCCTATAAA

IpgD GGCGCAGAAGAAACCGGAAAGCACAAAGGTGTAAG

IpgD GTGATTTCATATCAAAGAGTTAAA

IpgD AATGGAGAAAGAAATAAGGGGATCGAAGCGCTTAATCGATTATATTTACAGAACCAAACA

IpgD TTAACAGGAAAAAGCCTTTTGTTTGCGC

IpgD ATAAAGTTGGCAGGTGGTGATACGTCAAAAATTAAAGCCATGATGGAACGATTAGATACC

IpgD TATAAACTTGGTGAAGTTAATAAAAGACATATTAATGAGCTTAATAAAGTAATAAGTGAA

IpgD GAGATAAGAGCACAGCTAGGCATTAAA

IpgD ATAAAAAGGAATTGCAAACGAAAATAAAACAA

IpgD ATATTTACTGATTATTTAAATAATAAGAATTGGGGACCGGTC

IpgD CCTGCCTCTCATATGAAGATAGGGAATAAA

IpgD	AATATATTTGTCAAAGAGTATAATGGAAAAGGAATTTGTTGTGCTTCTACGA
IpgD	CATATCGCGAATATGTGGCTTTCAAAAGTGGTGGATGA
IpgD	TCGGGTATCAGACATGGAGTTATATC
IpgD	GCCTATGGATTGAAAAAAAACTCATCTGAAAGA
IpgD	GCTGTTGCTGCTCGTAATAAAGCTGAGGAGTTAGTAAGCGCAGCATTATATAGCAGACCA
IpgD	GAGTTATTATCACAGGCTTTGTCTGGTAAAACAGTAGATTTAAAGATTGTTTCGACTTCT
IpgD	AGTTTAACCGGGGGAGAGGAGAG
IpgD	GCATTAAAAGGGTTAAACTCTAAAAGGGGG
IpgD	GCCAACGAAATTATTAATTCGGAATAGT
IpgD	GATGGTCTTCTGAAAGAAGTAA
IpgD	CGTTAATCTAAAAGTGGTGACATTTAACTTTGGTGTA
IpgD	AATGAATTAGCGCTAAAAATGGGCTTAGGCTGGAGGAATGTTGACAAGCTTAATGATGAA
IpgD	TCAATATGTTCTTTGTTGGGGGATAATTTCCTCAAAAATGGTGTGATTGGAGGTTGGGCT
IpgD	GCAGAAGCAATAGAAAAAAATCCACCATGTAAGAATGATGTGATATATCTGGCTAACCAG
IpgD	TTACAAAAAAATGATAATGGAGAGCCGTATAAATTG
IpgD	TTACAAAAAAATGATAATGGAGAGCCGTATAAATTG
IpgD	AGTGGGAAGGACAGAACAGGTATGCAGGATGCTGAAATTAAAAGAGAAATAATAAGAAAA
IpgD	TAAATTATCCTCAGAAGAGAAAAGATTA
IpgD	TTTTCTACTATTCTAATGAATAGTGGTAATATGGAAATCCAAGAGATGAATACTGGTGTT

IpgD ATGAAAAAATTGCCGCTATCTTCGTTAGAGCTATCTTATTCTGAA

IpgD AAAATATGGAATATGGTGAAAGGGTATTCGTCATTTGTA

MxiC ATGCTTGATGTTAAAAATACAGGAGTTTTTAGCTCTGCATTCATTGATA

MxiC ATGACAAATTCAGATGATGGAGATGAGACTGCTGATGCAGAGCTTGATTCTGGCTTGGCT

MxiC AATAGCAAGTATATTGACTCATCTGATGAGATGGC

MxiC AGAAGAGACCTTGAGAAACTGAAAGGAA

MxiC GGGGAAGAAGATGAAATTAATCACAAGATTTTTGATTTAAAGAGAAC

MxiC GGATTTTATAGACAGACTAAAGAGATATTTTAAAGATCCAAG

MxiC CAAGTCTTAGCATTAAGGGA

MxiC CTTTTGAATGAAAAAGATCTTACTGCTGAACAAGTCGAA

MxiC ATTAACTAAAATTATTAATGAGATAATATCAGGTAGTGAAAAAAGTGTTAATGCTGGA

MxiC ATAAATTCAGCTATACAGGCTAAATTATTTGGCAACAAAATGAAACTTGAACC

MxiC GCATGTTATCGTGGTTTTATCATGGGGAACA

MxiC GAATGGCTTGGTAATTTTGGTTTTAATCACAG

MxiC CATACAATTGTGAATTTTGTAGAGCAG

MxiC TCACTGATTGTAGACATGGATTCTGAGAAACCGAGCTGTAAT

MxiC GTGTTATCTAAATTAATTGCAATTAAGATG

MxiC AAACTGGAATCCTCAAGCTTGCTAAAAGATGGC

MxiC ACTTTATTATATATTTTTCAATATCCAAGTGAAAGTGAGCAAATTCTTACTTCTGTTATA

MxiC AGCCAGTCATGAGGATTCTGTAGTGTATCAAACATATCTATCTTCTGTT

MxiC AATGAAAGTCCTCATGATATATTTAAAAGTGAAAGTGAAAGAGAAATTGCGATCAATATT

MxiC CTACGAGAGCTTGTCACAAGTGCATACAAGAAAGAGCTTTCTAGA

OspB ATGGGACAACAAATACCAAGAGTATTTAAGAACAAGATGTTATATGATTATGTTTTT

OspB AAAAATGAAAAAAGTAAAAATGATTTTCTAAAAATGGCTGAATCATGGCTACCACAGAGT

OspB TAGTAATAAATAATGATGATGACGCATTGAATGCTGCTGCTTATTTTTCTGTA

OspB ATTCTTGGGCACGGTAGTCCTGGTTCTCATCAATTAGGCCTTGGTTCGGA

OspB GTACAAACAATCATTTCAAGAATGAAAGACTGTGGTATTCTAAATGTGAAAGATATCCGT

OspB TTTACTTCATGCGGCTCCGCTGATAAAGTGGCTCCTAAA

OspB GAAAGTCTTTCTTGTATCCTTAACTCTCTGCCTTTTTTTAAGGAAAAAGAATCTTTGCTA

OspB GAGCAGATAAAAAAACACCTTGAAAACGATGAGTCATTGAGTGATGGTCTAAAAATATCC

OspB CACTATGGTCAAGAGCTTTTTCCCTACTCACATTATCGT

OspB TCAACTTCAATTCCTGCTGATCCGGAGCATACAGTAAAAAGAAGCTCTCAGAAAAAGACT

OspB TTTATTATTAATAAAGAACTGGATTAG

OspB GGCTATCATGGATATGGAGT

OspB

ATGGGACAACAAATACCAAGAGTATTTAAGAACAAGATGTTATATGATTATGTTTTTAAAAATGAAAAAAGTAAAAATGATTTTCTAAAAATGGCTGAATCATGGCTACCACAGAGTGAACCA

OspB

TAGTAATAAATAATGATGATGACGCATTGAATGCTGCTGCTTATTTTTCTGTAAAAAAAAGCGAAAATAAAAACAGTAAACGATACTGATTTTAAAGAGTATAATAAGGTTTATATTCTTGGGCACGGTAGTCCTGGTTCTCATCAATTAGGCCTTGGTTCGGA

OspB
CTTATTGATGTACAAACAATCATTTCAAGAATGAAAGACTGTGGTATTCTAAATGTGAAAGATATCCGTTTTACTTCATGCGGCTCCGCTGATAAAG
TGGCTCCTAAA

OspB
ATTTTAACAATGCCCCTGCTGAAAGTCTTTCTTGTATCCTTAACTCTCTGCCTTTTTTTAAGGAAAAAGAATCTTTGCTAGAGCAGATAAAAAAACAC
CTTGAAAACGATGAGTCATTGAGTGATGGTCTAAAAATATCCGGCTATCATGGATATGGAGT

OspB
CACTATGGTCAAGAGCTTTTTCCCTACTCACATTATCGTTCAACTTCAATTCCTGCTGATCCGGAGCATACAGTAAAAAGAAGCTCTCAGAAAAAGA
CTTTTATTATTAATAAAGAACTGGATTAG

OspF ATGCCCATAAAAAAGCCCTGTCTAAAACTAAATTTAGATTCTTTGAATGTTGTGAGGTCT

OspF GAAATACCGCAGATGCTTTCTGCGAATGAAAGATTGAAAAATAACTTCAATATATTGTAC

OspF AACCAAATACGGCAATACCCAGCGTACTATTTCAAAGTAGCATCGAACGTGCCAACTTAC

OspF TCTGATATATGTCAATTTTTTTCTGTCATGTACCAAGGATTCCAGATTGTGAACCATAGT

OspF GGAGATGTGTTTATTCATGCATGTCGTGAAAATCCTCAGAGTAAGGGGGATTTTGTTGGG

OspF GACAAGTTTCATATTAGTATAGCTAGGGAGCAAGTTCCTCTGGCATTCCAAATTTTGTCT

OspF GGTTTATTATTTTCAGAGGACAGTCCTATAGATAAATGGAAGATAACTGATATGAATCGA

OspF GTTTCTCAGCAATCTCGTGTGGGGATAGGAGCTCAGTTTACGCTATATGTAAAATCAGAT

OspF CAGGAATGCTCGCAATATAGTGCTTTATTACTTCATAAAATACGACAATTTATAATGTGT

OspF CTCGAGTCTAATCTATTAAGAAGCAAAATCGCTCCCGGGGAGTATCCGGCGTCAGATGTT

OspF AGACCTGAAGACTGGAAATATGTCAGCTATCGTAATGAACTACGAAGCGATCGAGATGGA

OspF AGTGAAAGGCAAGAGCAAATGTTACGAGAGGAACCATTTTATCGTTTGATGATAGAGTAG

OspF

ATGCCCATAAAAAAGCCCTGTCTAAAACTAAATTTAGATTCTTTGAATGTTGTGAGGTCTGAAATACCGCAGATGCTTTCTGCGAATGAAAGATTGA
AAAATAACTTCAATATATTGTACAACCAAATACGGCAATACCCAGCGTACTATTTCAAAGTAGCATCGAACGTGCCAACTTACTCTGATATATGTCAATTTTT
TTCTGTCATGTACCAAGGATTCCAGATTGTGAACCATAGTGGAGATGTGTTTATTCATGCATGTCGTGAAAATCCTCAGAGTAAGGGGGATTTTGTTGGGGA
CAAGTTTCATATTAGTATAGCTAGGGAGCAAGTTCCTCTGGCATTCCAAATTTTGTCTGGTTTATTATTTTCAGAGGACAGTCCTATAGATAAATGGAAGATA
ACTGATATGAATCGAGTTTCTCAGCAATCTCGTGTGGGGATAGGAGCTCAGTTTACGCTATATGTAAAATCAGATCAGGAATGCTCGCAATATAGTGCTTTA
TTACTTCATAAAATACGACAATTTATAATGTGTCTCGAGTCTAATCTATTAAGAAGCAAAATCGCTCCCGGGGAGTATCCGGCGTCAGATGTTAGACCTGAA
GACTGGAAATATGTCAGCTATCGTAATGAACTACGAAGCGATCGAGATGGAAGTGAAAGGCAAGAGCAAATGTTACGAGAGGAACCATTTTATCGTTTGAT
GATAGAGTAG

OspG ATGAAAATAACATCTACCATTATTCAAACACCTTTTCCATTTGAGAATAATAATTCTCAT

OspG GCTGGCATAGTAACGGAGCCCATTCTCGGTAAGTTAATAGGTCAGGGGTCGACAGCAGAA

OspG ATCTTTGAAGATGTGAATGATTCATCTGCTTTGTATAAAAAGTATGATCTTATTGGCAAC

OspG CAGTACAATGAGATTCTGGAAATGGCTTGGCAAGAATCTGAGCTTTTTAATGCTTTTTAT

OspG GGCGATGAAGCATCCGTTGTTATACAGTATGGCGGAGATGTGTACCTCCGAATGCTGCGC

OspG GTGCCTGGGACTCCCCTTAGTGACATTGATACAGCTGATATCCCTGATAATATAGAGAGC

OspG CTTTATCTACAGTTGATATGTAAATTGAATGAGTTGAGTATAATCCATTACGATCTTAAT

OspG ACAGGTAATATGCTGTATGATAAAGAAAGTGAAAGTTTATTCCCAATAGATTTTCGCAAT

OspG ATTTATGCTGAATATTACGCTGCAACCAAAAAAGATAAAGAGATTATCGACCGACGATTA

OspG CAAATGCGTACAAATGATTTTTATTCGTTATTAAACAGGAAATATTTATAG

OspG

TAACGGAGCCCATTCTCGGTAAGTTAATAGGTCAGGGGTCGACAGCAGAAATCTTTGAAGATGTGAATGATTCATCTGCTTTGTATAAAAAGTATGA
TCTTATTGGCAACCAGTACAATGAGATTCTGGAAATGGCTTGGCAAGAATCTGAGCTTTTTAATGCTTTTTATGGCGATGAAGCATCCGTTGTTATACAGTAT
GGCGGAGATGTGTACCTCCGAATGCTGCGCGTGCCTGGGACTCCCCTTAGTGACATTGATACAGCTGATATCCCTGATAATATAGAGAGCCTTTATCTACAG
TTGATATGTAAATTGAATGAGTTGAGTATAATCCATTACGATCTTAATACAGGTAATATGCTGTATGATAAAGAAAGTGAAAGTTTATTCCCAATAGATTTTC

GCAATATTTATGCTGAATATTACGCTGCAACCAAAAAAGATAAAGAGATTATCGACCGACGATTACAAATGCGTACAAATGATTTTTATTCGTTATTAAACA GGAAATATTTATAG

Spa33 AAGCAACTCTGCGAGCGTTTT

Spa33 CGATTCAGACATTCAAAAATAAATTTACAGGCAGTGAAAGTTTA

Spa33 GTCACTCTTACTTCCGTGTGTGG

Spa33 CGATTCAGACATTCAAAAATAAATTTACAGGCAGTGAAAGTTTAGTCACTCTTACTTCCGTGTGTGG

Spa33 ATTCGTATTGATACATTATCTTTTTTG

Spa33 AAAAAAAAATACGAGGTATTTTCAGGATTTTCTACACAAGAATCTTTACTGCATTTATCA

Spa33 ATTCGTATTGATACATTATCTTTTTTGAAAAAAAAATACGAGGTATTTTCAGGATTTTCTACACAAGAATCTTTACTGCATTTATCA

Spa33 AAATGTGTCTTTATAGAGTCGTCATCTGTATTTTCGATTCCAGAACTGTC

Spa33 GATAAGATTACTTTCCGGATCACGAATGAAATCCAGT

Spa33 ACTTTCCGGATCACGAATGAAATCCAGT

Spa33 TGCAACTACTGGGAGTCATTTATGCTGTTTT

Spa33 TCCTCTTCTTTAGGTATTATTTAT

Spa33 TGCAACTACTGGGAGTCATTTATGCTGTTTTCCTCTTCTTTAGGTATTATTTAT

Spa33 TTGACAAGATGCCGGTATTACGTAATCAAGTTTCTCTTGA

Spa33 ATTGCATCATCTTTTAGAGTTTTGCTTAGGTTCATCTAATGT

Spa33 GCTACTTTAAAAAGAATTCG

Spa33 ACTGGTGATATAATCATAGTTCAGAAACTTTATAATTTATTA

Spa33 TTGTGTAATCAAGTTATTATTGGGGATTATATTGTGAATGATAATAATGAGGCAAAAATT

Spa33 AATCTGTCAGAAAGTAATGGTGAGTCAGA

Spa33 ACTGGTGATATAATCATAGTTCAGAAACTTTATAATTTATTATTGTGTAATCAAGTTATTATTGGGGATTATATTGTGAATGATAATAATGAGGCAAAAATTAATCTGTCAGAAAGTAATGGTGAGTCAGA

Spa33 CACACAGAAGTTTCTTTGGCATTATTCAAT

Spa33 TATGATGATATCAATGTAAAAGTGGACTTTA

Spa33 CACACAGAAGTTTCTTTGGCATTATTCAATTATGATGATATCAATGTAAAAGTGGACTTTA

Spa33 ATGACAATCAATGAACTAAAAATGTATGTAGAAAACGAATTATTCAAGTTTCCCGATGACATAGTTAAACAT

Spa33 GTAAATATTAAAGTAAATGGTTCTTTGGTTGGGCATGGGGAACTTGTTTCTATTGAGGAT

Spa33 GGTTATGGTATCGAGATTAGTTCTTGGATGGTAAAGGAGTAA

Spa33 GTAAATATTAAAGTAAATGGTTCTTTGGTTGGGCATGGGGAACTTGTTTCTATTGAGGATGGTTATGGTATCGAGATTAGTTCTTGGATGGTAAAGGAGTAA

VirA ATGCAGACATCAAACATAACTAAC

VirA ATGAAAGAAATGACTCATCATGGATGTCAACTGTC

VirA ACAGAAGTAAGTTGGAATAAATTGAGTTTTTGTGAC

VirA ATATATTCTCCACACGAAACTCTAGCCGAAAAA

VirA CTAATGGATAGTTTCTCACCATCACTATCACAAGATAAAATGGACGGAGAGTTTGCCCAC

VirA GCAAACATAGATGGCATTTCAATACGGCTATGCCTTAACAAAGGCATATGTTCGGT

VirA TATCTTGATGGAGATAAAATACAATCCACTCAACTTTCATCAAAAGAATATAATAACTTG

VirA AAACAATTCAACTTAGGAAAAGTACATACTATTACAGCTCCGTTAAGTTCACTCCCCCC

VirA	GTATCAGGGAATTTTAAAACTCACAAGCCTGCACCAGAAGTTATTGAAACAGCCATTAAT
VirA	ACATCGATAATACCAA
VirA	CGTTAAAGACACTGATTTTAAT
VirA	AGTGTTTGGCATGATATTTACAGAGATATT
VirA	TATTTCAACAATATTGAAATACC
VirA	AGATCTAATTAATGAGTTAGGA
VirA	ATTGATAGCAAAAAAGAGCTTCAAATGCTAAGTTACAACCAA
VirA	AAAATAATCAATTCAAACTTCCCCCAACAAGACCTATGTTTTCAAACAGAAAAACTTTTG
VirA	TTTACTTCACTCTTTCAAGACCCTGCATTTATATC
VirA	GCACTAACATCTGCATTCTGGCAA
VirA	TCTCTTCACATCACGTCTTCCTCTGTGGAACACATATATGCCCAAAT
VirA	ATAGAAAACAGGCTTAATTT
VirA	ATGCCTGAACAACGAGTTATTAACAATTGTGGACATATT
VirA	AGTTGTCCCTAAAAACGACACTGCAATCTCTGCCTCTGG
VirA	GCTTACGAAGTTAGCTCATCAATATTACCATCGCA
VirA	ATAACATGTAATGGTGTTGGTATT
VirA	AATAAAATAGAAACAAGCTATCTCGTTCATGCTGGAACACTTCCAAGCAGTGAAGGGCTT
VirA	CGCAATGCTATCCCTCCAGAAAGTCGTCAAGTATCATTTGCCATCATATCTCCTGATGTT
VirB	ATGGTGGATTTGTGCAACGACTTGTTAAGTATAAAGGAAGGCCAAAAGAAAGAG

VirB CTCCATTCTGGTAATAAAGTTTCCTTTATCAAAGC

VirB AAGATTCCTCATAAAAGGATCCAA

VirB GATTTAACCTTCGTCAACCAAAAAACGAATGTACGCGATCAAGAATCCCTAACAGAAGAA

VirB TAGCCGATATCATAAAAACTATAAAGCT

VirB CAACAATTCTTCCCTGTAATAGGAAGG

VirB GAGATTGATGGTAGAATTGAAATTCTGGATGGCACTCGTAGAAGAGCATCTGCAATATAT

VirB GCAGGAGCAGATCTTGAAGTTCTATATTCAAAAGAATATATATCTACTCTTGATGCCAGA

VirB AAACTAGCAAACGATATACAAACAGCAAAAGAGCATAGCATCCGAGAACTTGGTAT

VirB CTTAATTTTCTGAAAGTATCAGGGATGTCCTATAAAGACATAGCCAAAAAAGAGAATCTG

VirB TCTCGCGCGAAAGTCACTCGTGCCTTTCAGGCAGCAAGCGTTCC

VirB CTATTTCCAATCGCGTCAGAACTTAACTTTAATGACTACAAGATATTATTCAATTATTAT

VirB AAAGGACTTGAAAAGGCTAATGAATCTCTTAGTTCTACACTACCAATATTAAAGGAAGAA

VirB TGCCCCCGGACATATATAAAAAAGAAATTTTAAACATC

VirB ATAAAGAAAAGCAAAAACAGAAAGCAAAACCCTTCGCTAAAAGTTGACTCGTTATTTATT

VirB TCTAAAGACAAACGGACTTACATAAAAAGAAAAGAGAATAAAACAAACAGAACTCTAATA

VirB TTTACATTATCTAAAATAAACAAAACAGTTCAGAGAGAAATAGATGAAGCTATTCG

VirB ATAATTTCTCGCCATCTATCGTCTTCA

VirF ATGATGGATATGGGACATAAAAACAAAATAGATATAAAGGTTCGCTTGCATAACTATATT

VirF ATTTTATATGCAAAAAGGTGTTCAATGACGGTTAGCTCAGGCAATGAAACTTTGACTATC

VirF ATGAAGGGCAAATTGCTTTTATAGAG

VirF GAAATATACAAATAAACGTCTCCATAAAAAAA

VirF ATCCATTTGAGATTATAAGCCTTGACAGAAATTTATTATTAAGCATT

VirF ATTAGAATAATGGAACCAATTTATTCATTTCAACACTCCTATTCTGA

VirF CCTCCTCTCTGAGGAGGAGGTTTCTATCGATTTGTTCAAATCT

VirF GGCAAAAGAAAGATCTATAGTTTAGCTTGCCTTTTATCAGCT

VirF GTTTCTGATGAGGAAGCTTTATATACTTCGATATCGATAGCTTCTTCTCTTAGTTTTTCT

VirF GATCAGATAAGGAAGATTGTTGAAAAAAACATCGAGAAGAGATGGCGTCTTTCTGATATT

VirF TCAAATAACTTGAATTTATCAGAAATAGCTGTTAGAAAACGA

VirF ACATTTCAACAAATCCTTCTTGATATTCGCATGCATCATGCAGCAAAGCTTTTATTGAAT

VirF CAAAGCTATATTAATGATGTATCAAGACTTATCGGAATATCAAG

VirF ATAAGGAAATTTAATGAATATTATGGTATAACTCCAAAGAAATTTTACTTATATCATAAAAAATTT

VirF
GGCAAAAGAAAGATCTATAGTTTAGCTTGCCTTTTATCAGCTGTTTCTGATGAGGAAGCTTTATATACTTCGATATCGATAGCTTCTTCTCTTAGTTTT
TCTGATCAGATAAGGAAGATTGTTGAAAAAAACATCGAGAAGAGATGGCGTCTTTCTGATATTTCAAATAACTTGAATTTATCAGAAATAGCTGTTAGAAAA
CGA

VirF TGGAGAGTGAAAAATTAACATTTCAACAAATCCTTCTTGATATTCGCATGCATCATGCAGCAAAGCTTTTATTGAATAG

Supplementary Table S1. Components of the URA rules (first generation algorithms) for siRNA designing

Name of the rule for siRNA designing	Description
Ui-Tei	A or U at the 5′ end of the sense strand
	G or C at the 5′ end of the antisense strand
	Length of GC repeats less than 9 nucleotides
	Duplex End A or U differential >0
	No U present at position 1
Amarzguioui	High affinity of binding for the 5′ end of the sense strand
	A present at position 6
	Low affinity of binding for the 3′ end of the sense strand
	GC content: 30-52% (1 point)
	Three or more A/U at position 15-19 of sense strand (1 point)
Reynolds	Minimum internal stability at the target site (Tm > −20 °C)
	U at position 10 of the sense strand (1 point)
	A at position 3 of the sense strand (1 point)
	A at position 19 of the sense strand (1 point)
	No G at position 13 of the sense strand (1 point)

*Footnotes: Threshold score for efficient siRNA ≥ 6
