# Supplementary material for: A Comprehensive Computational Investigation into the Conserved Virulent Proteins of Shigella species Unveils Potential Small-Interfering RNA Candidates as a New Therapeutic Strategy against Shigellosis
Source: Molecules. 2022 Mar 17;27(6):1936. doi: 10.3390/molecules27061936 (PMC8950558; doi:10.3390/molecules27061936)
Supplement: Supplementary file 1 [file molecules-27-01936-s001.zip › Supplementary Table S1. Component of URA rules used for siRNA designing.pdf]

Supplementary Table S1. Components of the URA rules (first generation algorithms) for siRNA designing

Name of the rule for siRNA designing	Description
Ui-Tei	A or U at the 5′ end of the sense strand
	G or C at the 5′ end of the antisense strand
	Length of GC repeats less than 9 nucleotides
	Duplex End A or U differential >0
	No U present at position 1
Amarzguioui	High affinity of binding for the 5′ end of the sense strand
	A present at position 6
	Low affinity of binding for the 3′ end of the sense strand
	GC content: 30-52% (1 point)
	Three or more A/U at position 15-19 of sense strand (1 point)
Reynolds	Minimum internal stability at the target site (Tm > −20 °C)
	U at position 10 of the sense strand (1 point)
	A at position 3 of the sense strand (1 point)
	A at position 19 of the sense strand (1 point)
	No G at position 13 of the sense strand (1 point)

*Footnotes: Threshold score for efficient siRNA ≥ 6
